# Achilles tendon allograft augmentation for biomechanical residual limb pain following below-knee amputation

**DOI:** 10.1016/j.jvscit.2026.102477

**Published:** 2026-08-24

**Authors:** Abigail Teitelbaum, Stephanie Taiberg, John M. Felder

**Affiliations:** a Corewell Health William Beaumont University Hospital, Royal Oak, MI; b Rosalind Franklin University of Medicine and Science Chicago Medical School, Chicago, IL

**Keywords:** Achilles tendon allograft, Amputation stump revision, Below-knee amputation, Residual limb pain

## Abstract

Chronic residual limb pain after below-knee amputation may result from inadequate distal soft-tissue padding, leading to poor prosthetic tolerance and impaired function. We describe a novel technique using Achilles tendon allograft augmentation to treat biomechanical stump pain. Four patients underwent revision below-knee amputation with Achilles tendon allograft placement between August 2023 and February 2025. The mean age was 51 years, and the average follow-up was 4 months. All experienced complete pain resolution and successful return to prosthetic use without complications or additional revision. Achilles tendon allograft augmentation is a simple, effective option for carefully selected patients.

Below-knee amputation (BKA) is common in patients with trauma, infection, vascular disease, and diabetic complications. Despite advances in prosthetic design and rehabilitation, many patients have chronic residual limb pain that limits prosthetic tolerance and functional recovery.^1^ While neuropathic etiologies such as neuroma and phantom limb pain are well recognized, biomechanical pain from inadequate soft-tissue padding over the distal tibia is a potential cause of pain at rest and with ambulation on a prosthesis.

Insufficient soft-tissue coverage can cause focal pressure, recurrent skin breakdown, poor stump contour, and pain at rest and during ambulation.^2^ Current revision strategies for painful BKA stumps include scar revision, myodesis revision, targeted muscle reinnervation, regenerative peripheral nerve interfaces, soft-tissue flap reconstruction, and reamputation.^3^ However, few techniques specifically address inadequate durable padding over bony prominences.

Achilles tendon allograft is widely used in reconstructive surgery for its strength, durability, and predictable incorporation.^4^, ^5^, ^6^ We present a novel application for distal stump padding in patients with chronic biomechanical pain after BKA.

## Methods

### Study design

A retrospective case series evaluated four patients who underwent revision BKA with Achilles tendon allograft augmentation between August 2023 and February 2025.

### Patient selection

Four patients underwent revision surgery for chronic residual limb pain attributed to inadequate soft-tissue padding over the distal tibia (Fig 1). All reported pain at rest and during prosthetic use, with impaired ambulation and difficulty with prosthetic fitting.

**Fig 1:**
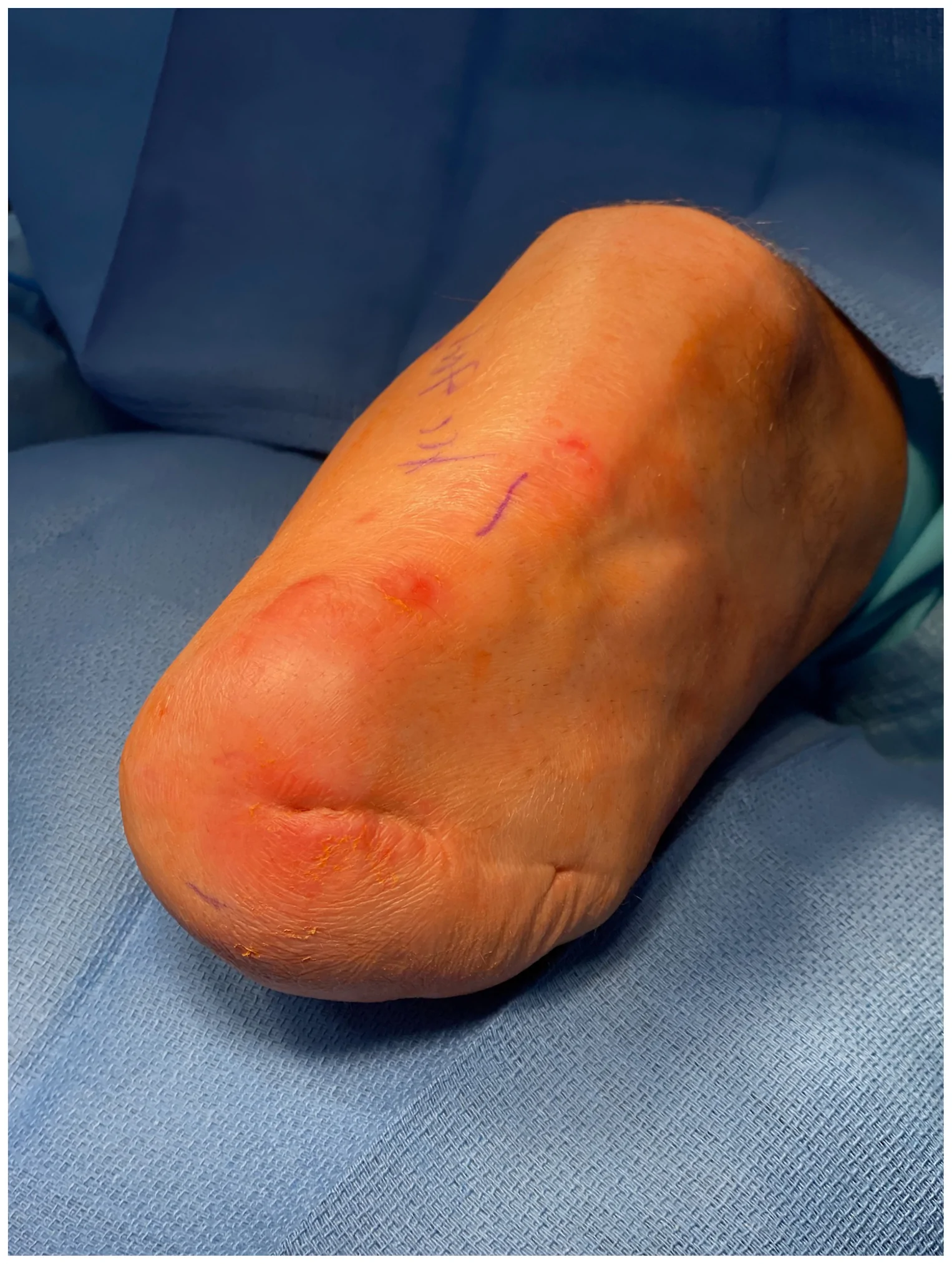
Preoperative appearance of a painful below-knee amputation (BKA) stump demonstrating inadequate distal soft-tissue padding and contour deficiency over the distal tibia.

Patients underwent clinical evaluation to exclude neuroma, phantom limb pain, active infection, and vascular insufficiency. Laboratory studies and imaging were obtained selectively when the history or physical examination raised concern for infection.

### Surgical technique

Achilles tendon allograft is inserted into a subcutaneous tunnel overlying the distal tibial stump, using counterincisions to avoid wound breakdown at the old scar over the bone end (Fig 2, *A* and *B*). This augments distal padding while minimizing soft-tissue compromise.

**Fig 2:**
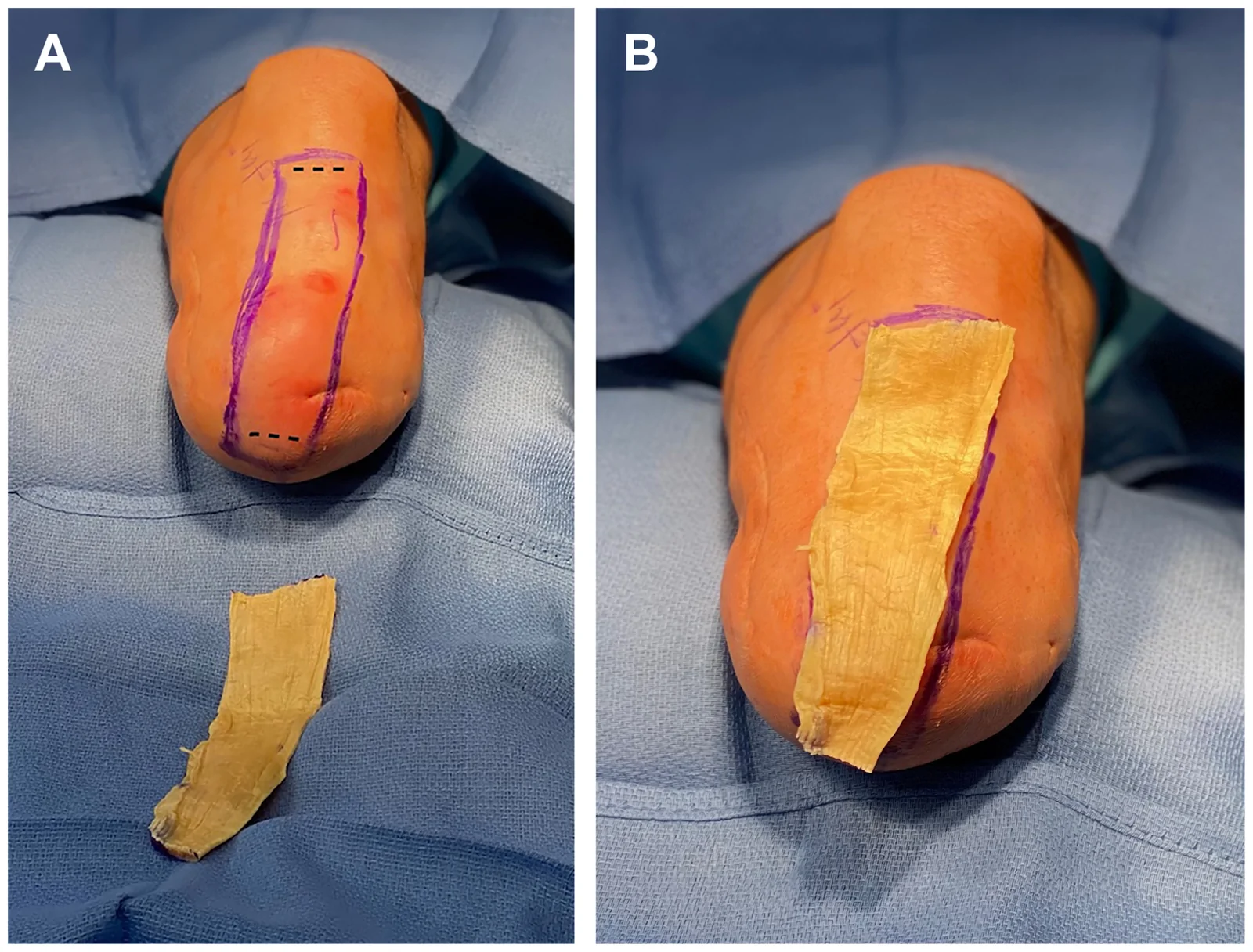
Preoperative surgical planning for Achilles tendon allograft augmentation. **A,** Planned proximal and distal counterincisions used to create a subcutaneous tunnel while avoiding the distal weight-bearing surface of the stump. **B,** Achilles tendon allograft positioned externally to demonstrate its final orientation over the distal tibia following tunnel placement, providing additional soft-tissue padding and contour restoration.

After local anesthetic is infiltrated along the planned trajectory, 3- to 4-cm transverse counterincisions are made 3 to 5 cm proximal and distal to the tibial bone stump. A subcutaneous tunnel is developed between the counterincisions using spreading dissection to elevate skin and subcutaneous tissue off the underlying fascia and bone. The pocket should be kept tight relative to the allograft shape to avoid migration.

The allograft is thawed and prepared on the back table by removing the calcaneal bone block, then tailored to the contour and dimensions of the residual limb defect. Where additional bulk is needed, the tendon can be folded onto itself and secured with 2-0 Vicryl sutures to increase padding and improve contour.

The graft is passed through the tunnel with tonsil clamps, positioned over the distal tibia, and secured to the deep fascia with interrupted 2-0 Vicryl sutures through the limited-access incisions. The allograft is positioned entirely within the subcutaneous plane and secured to the underlying fascia without tension, serving solely to augment distal soft-tissue bulk.

Incisions are closed with Prolene mattress sutures.

### Postoperative management

Patients remain non-weight bearing and resume prosthetic use after approximately 6 weeks. Prosthetic modification is performed by the treating prosthetist as clinically indicated.

### Data collection

Demographics, operative details, complications, pain outcomes, prosthetic tolerance, and need for additional revision were collected retrospectively.

## Results

The median patient age was 54 years (interquartile range, 42.5-59.5). Indications for the initial BKA were diabetic foot infection in two patients and traumatic injury in two patients. Median follow-up was 3.5 months (interquartile range, 3-5 months).

Three patients underwent isolated Achilles tendon allograft augmentation through counterincisions with subcutaneous tunneling. One additionally required distal tibial contouring with anterior cortical beveling and distal fibular shortening for significant bony prominence and recurrent wounds.

All patients reported complete pain resolution and resumed prosthetic use with improved tolerance. No postoperative complications, wound healing issues, graft-related complications, or additional revisions were observed during follow-up (Supplementary Video 1, online only, Fig 3, *A* and *B*).

**Fig 3:**
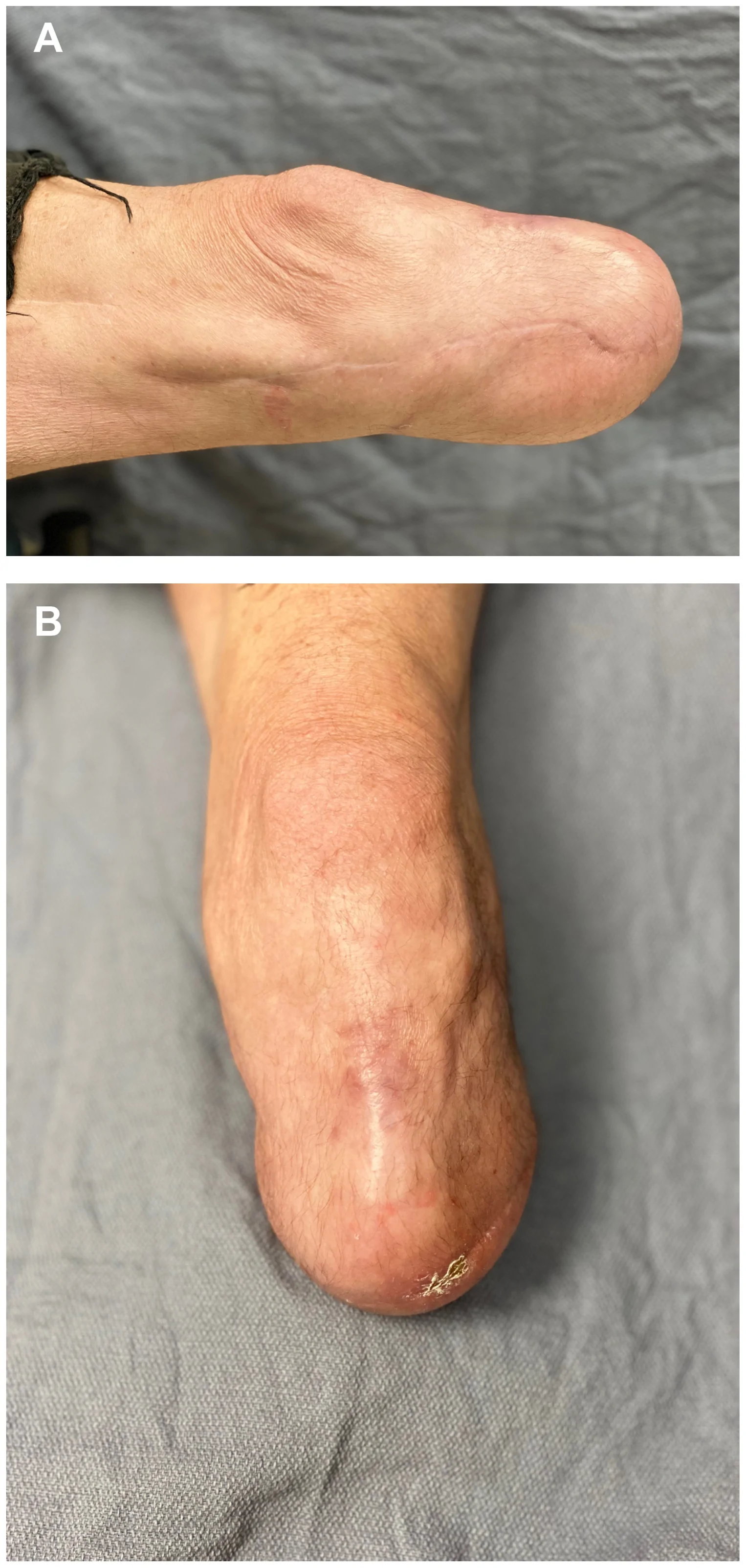
Four-month postoperative follow-up demonstrating successful augmentation of the distal residual limb. **A,** Lateral view showing restoration of soft-tissue contour over the distal tibia. **B,** Distal view demonstrating increased padding of the weight-bearing surface.

## Discussion

This novel technique is a simple approach to a common problem: painful BKA stumps caused by inadequate soft-tissue padding. It was inspired by the Achilles tenodesis to the tibia performed at the time of BKA—an important but frequently forgotten step intended to prevent this issue.^7^ It is best suited for patients with focal distal tenderness, inadequate soft-tissue coverage, pain exacerbated by prosthetic loading, and no alternative etiology.

A major strength of this technique is that it directly addresses the mechanical etiology of pain while avoiding proximal reamputation or bone shortening, both of which risk recurrent pain and altered ambulation mechanics. In the single patient requiring concomitant osseous revision, the anterior tibial cortex was beveled conservatively, and distal fibular shortening reduced focal prominence without altering limb length. However, osseous contouring alone would not have restored a durable weight-bearing surface, as the residual limb remained covered by a thin, attenuated soft-tissue envelope requiring augmentation with the Achilles tendon allograft.

The Achilles tendon allograft provides dense, robust tissue able to withstand the repetitive compressive forces of prosthetic ambulation; it is readily available and avoids donor-site morbidity. Its elongated geometry allows it to be tunneled into a precisely developed subcutaneous pocket and positioned to overlap the tibia precisely while minimizing disruption of the fragile distal soft-tissue envelope. The graft may also be folded onto itself for greater bulk where needed.

Using proximal and distal counterincisions away from the distal tibial prominence reduces the potential for wound healing complications, critical in medically complex patients.^8^ Similarly, a narrow subcutaneous tunnel allows stable graft positioning.

Proper index BKA technique should include a posterior flap myodesis or Achilles tenodesis to the anterior tibia to provide durable posterior coverage over the distal stump.^7^, ^8^, ^9^, ^10^ However, this step is frequently neglected. Even when these maneuvers are performed, atrophy and ptosis can develop over time, progressively thinning the distal envelope.^8^ The resulting tibial prominence is painful and can make tolerating a prosthesis difficult despite an otherwise well-healed amputation.

This study is limited by its small sample size, retrospective design, and short follow-up; long-term graft durability has not been established, and larger studies with longer follow-up are needed to define this technique's role in residual limb reconstruction. Nonetheless, the concept and mechanism are simple, and all patients had complete pain resolution and successful prosthetic use without complications or additional revision. These early results suggest that Achilles tendon allograft augmentation may be a simple, reproducible, and minimally invasive option for carefully selected patients with painful BKA stumps from inadequate distal soft-tissue padding. As rehabilitation advances continue to improve mobility and longevity among amputees, optimizing quality of life and function may become an increasingly important focus in amputation surgery.

## Conclusions

Achilles tendon allograft augmentation is a simple, safe, and promising option for biomechanical pain following BKA in patients with inadequate distal soft-tissue padding. By addressing inadequate distal padding, it provides pain relief and successful prosthetic use without donor-site morbidity. Further investigation is warranted to evaluate long-term outcomes and define its role within the broader reconstructive algorithm for painful residual limbs.

## Funding

None.

## Disclosures

None.

## Appendix (online only)

Video 1 Four-month postoperative examination demonstrating durable distal stump padding following Achilles tendon allograft augmentation. The patient exhibits painless palpation of the distal residual limb and successful restoration of a weight-bearing soft-tissue envelope.
